# Supplementary material for: High-resolution snapshots of human N-myristoyltransferase in action illuminate a mechanism promoting N-terminal Lys and Gly myristoylation
Source: Nat Commun. 2020 Feb 28;11:1132. doi: 10.1038/s41467-020-14847-3 (PMC7048800; doi:10.1038/s41467-020-14847-3)
Supplement: Supplementary file 1 — Supplementary information [file 41467_2020_14847_MOESM1_ESM.pdf]

SUPPLEMENTARY INFORMATION

High-resolution snapshots of *N*-myristoyltransferase in action illuminate a mechanism promoting N-terminal Lys and Gly myristoylation

C. Dian, I. Pérez-Dorado et al.

Supplementary Figures

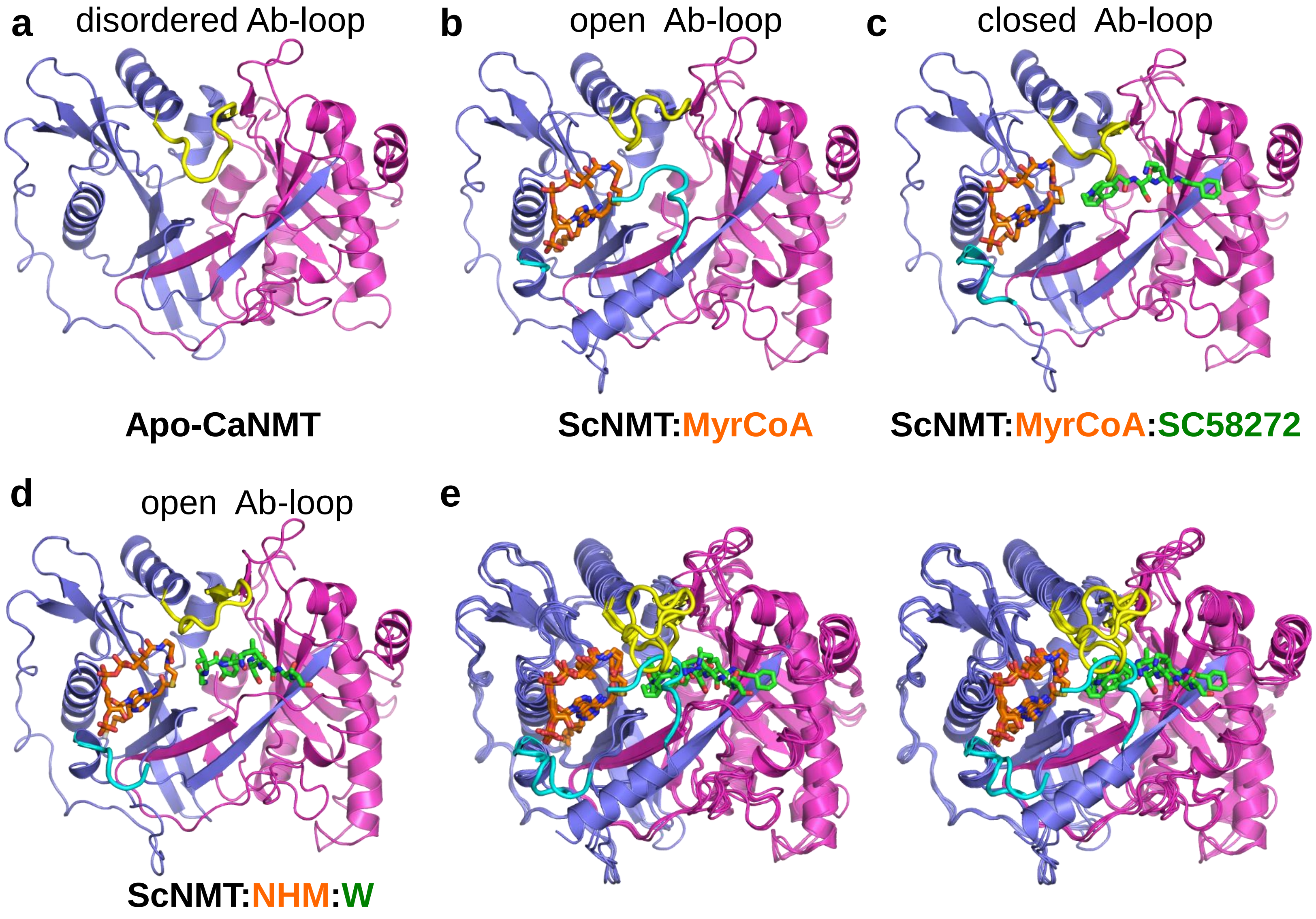

Supplementary Figure 1. Structures used to build the previously-accepted NMT catalytic mechanism
Overview of previously reported NMT crystal structures revealing the different conformations of both the B'A'- and Ab-loops. NMT main chains are displayed as ribbons. GNAT subdomains of NMTs are coloured in blue and pink, respectively. B'A' and Ab-loops are coloured in cyan and yellow, respectively. MyrCoA or NHM moieties are shown as orange sticks. Peptide substrate W and peptidomimetic inhibitor SC58272 are shown as green sticks. **a)** Crystal structure of apo CaNMT (PDB 1NMT, chain B)[1]. **b)** Crystal structure of ScNMT:MyrCoA binary complex (PDB 26PE, chain A)[2]. **c)** Crystal structure of ScNMT:MyrCoA:SC58272 ternary complex (PDB 2NMT)[3]. **d)** Crystal structure of ScNMT:MyrCoA:peptide W ternary complex (PDB 1IID)[4]. **e)** Stereoview of superimposed crystal structures shown in a, b, c and d.

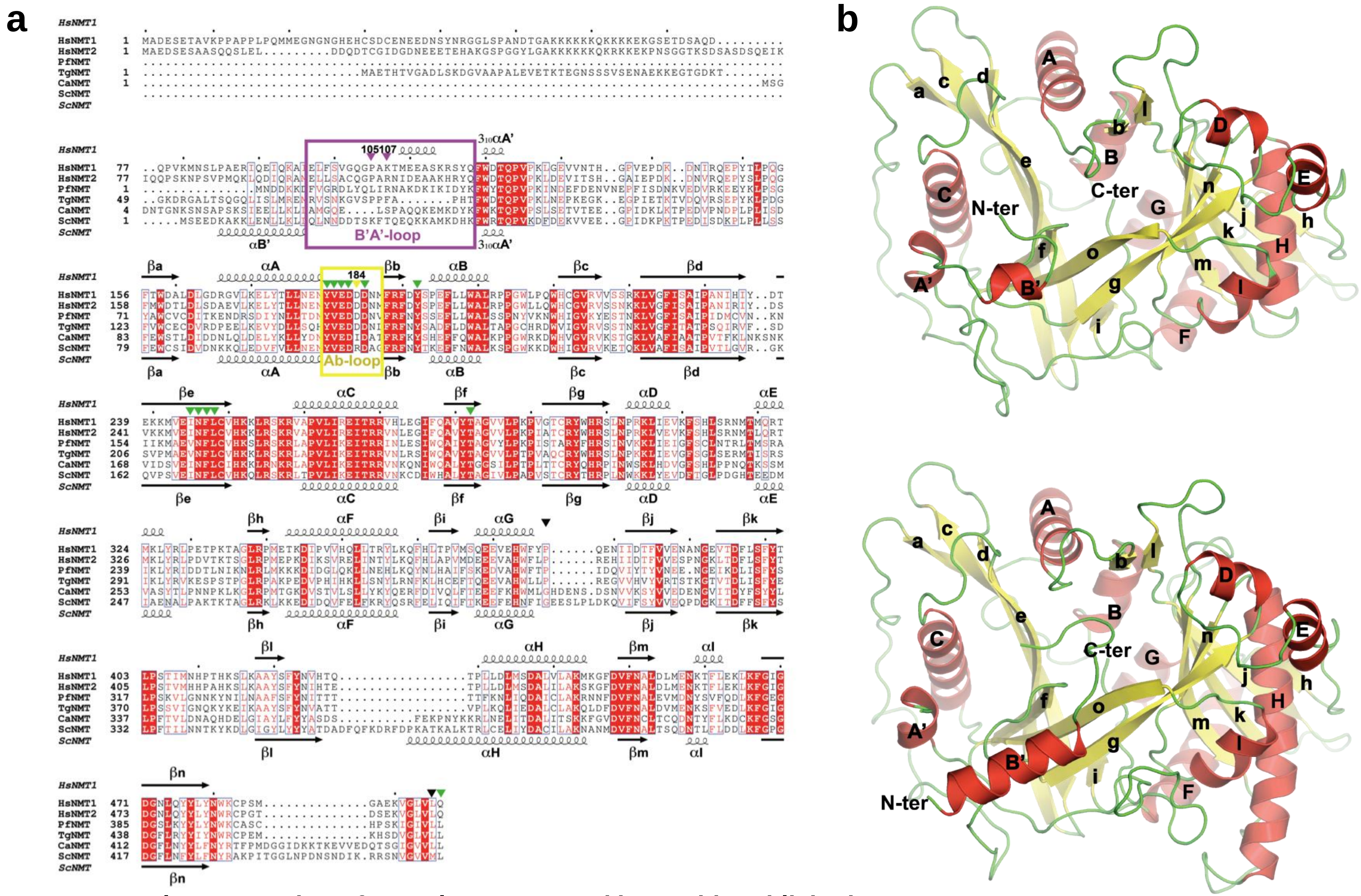

Supplementary Figure 2. Structural conservation of complexes reported here with published structures

a) Sequence alignment of NMTs with the corresponding secondary structure of HsNMT1:MyrCoA:X (top) and ScNMT:MyrCoA crystal structures (bottom, pdb code 1IID)[4] obtained with ESPript3 (http://espript.ibcp.fr,[5]). Magenta and yellow boxes indicate the positions of the B'A'- and Ab- loops, respectively. Residues identified in this study as important for the MYR mechanism are indicated with triangles. Green triangles define conserved residues, whereas yellow and magenta triangles denotes non-conserved ones, using B'A'- and Ab-loops colour coding. **b)** View of HsNMT1:MyrCoA:X (top) and ScNMT:MyrCoA (bottom)[4] folds labelled with secondary structure elements according to **a)**. Both crystal structures are displayed as ribbons. a-helix, b-strand, and loop are coloured in red, yellow and green, respectively.

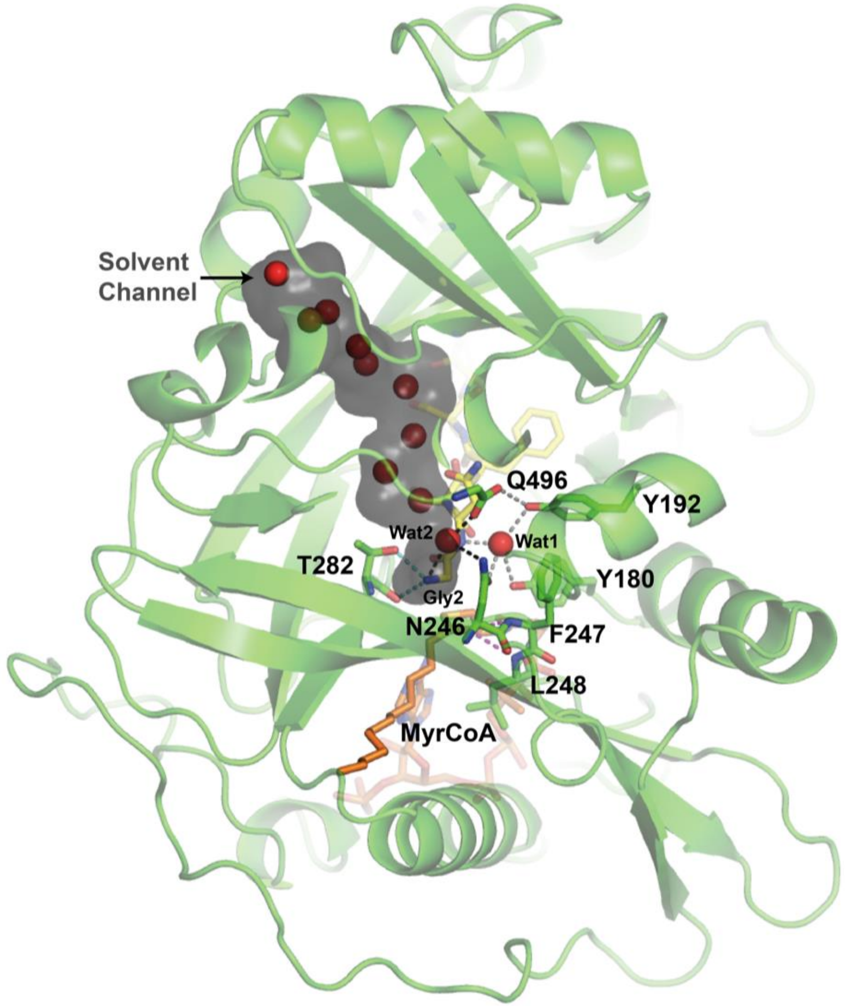

Supplementary Figure 3. Space filling cartoon representation of the water channel

View of the specific channel feeding the active site of HsNMT1 with water molecules in the HsNMT1:MyrCoA:X crystal structure. The solvent channel surface is shown as a grey surface. The HsNMT1 chain is displayed as a green ribbon, and selected amino acids are shown as green sticks. Substrate peptides (yellow) and MyrCoA and CoA moieties (both in orange) are shown as sticks. Hydrogen bond set1, linking Gly2 to Thr282 catalyst platform, are shown as cyan dashes. Wat1-mediated hydrogen bond set2, linking the carboxy terminus of Q496 to the hydroxyl groups of Tyr180/Tyr192 from the Ab-loop, amide group of substrate aa3, and amide of Asn246 are shown as grey dashes. Wat1-mediated hydrogen bond set3, linking the carboxy terminus of Gln496 to Gly2 ammonium, is shown as black dashes. Water molecules from the solvent channel are shown as red spheres. Hydrogen bonds involved in the "oxyanion hole" are displayed as a magenta dashed line. The H-bond between thiolate and amine from CoA is shown with violet dashes.

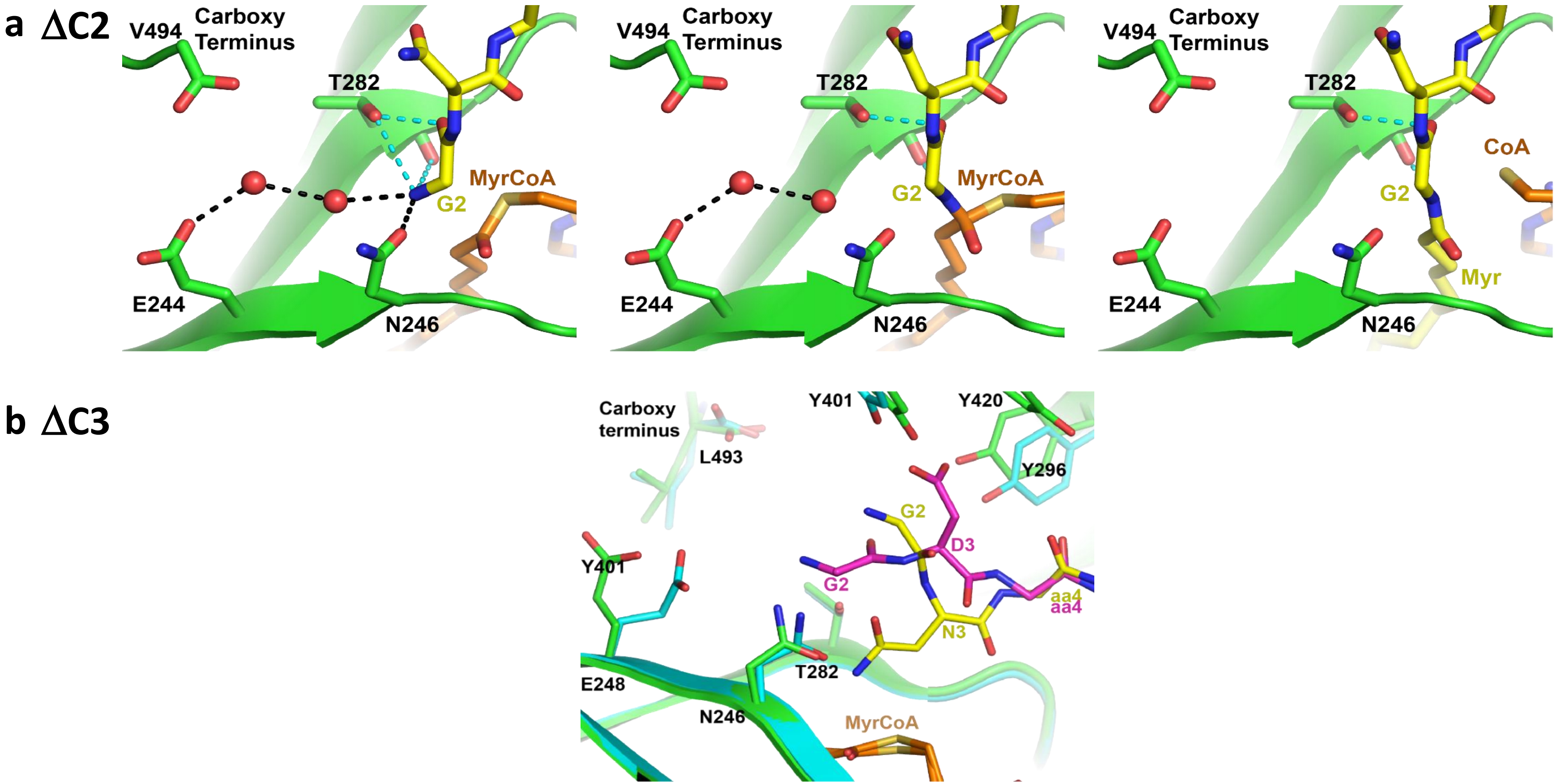

Supplementary Figure 4. Crystal structures of short C-terminal truncations of HsNMT1

View of C-terminal HsNMT1 truncations' active site. **a)** Snapshots of the MYR reaction trapped in crystal structure of HsNMT1ΔC2 (green) in complex with MyrCoA (orange) and peptide S (GlyAsnCysPheSerLysProArg; yellow) showing: substrates (left; conformation A and chain A), TI (middle; conformation B and chain A or conformation A and chain B), and products (right; conformation B and chain B) of the reaction. **b)** Superimposition of HsNMT1DC3 (cyan) in complex with MyrCoA (orange) and peptide S (GlyAsnCysPheSerLysProArg) with HsNMT1ΔC3 (green) in complex with MyrCoA (orange) and peptide T (GlyAspCysPheSerLysProArg) revealing a different conformation of the N-terminal extremity of substrate peptides in the enlarged cavity. The HsNMT1 chain is displayed as a ribbon, and selected amino acids are shown as sticks. Substrate peptides are shown as sticks. MyrCoA and CoA moieties shown as orange sticks. Hydrogen bond set1, linking Gly2 to T282 catalyst platform, is shown as cyan dashes. Wat-mediated hydrogen bond set linking the carboxylic group of E244 to Gly2 ammonium are shown as black dashes.

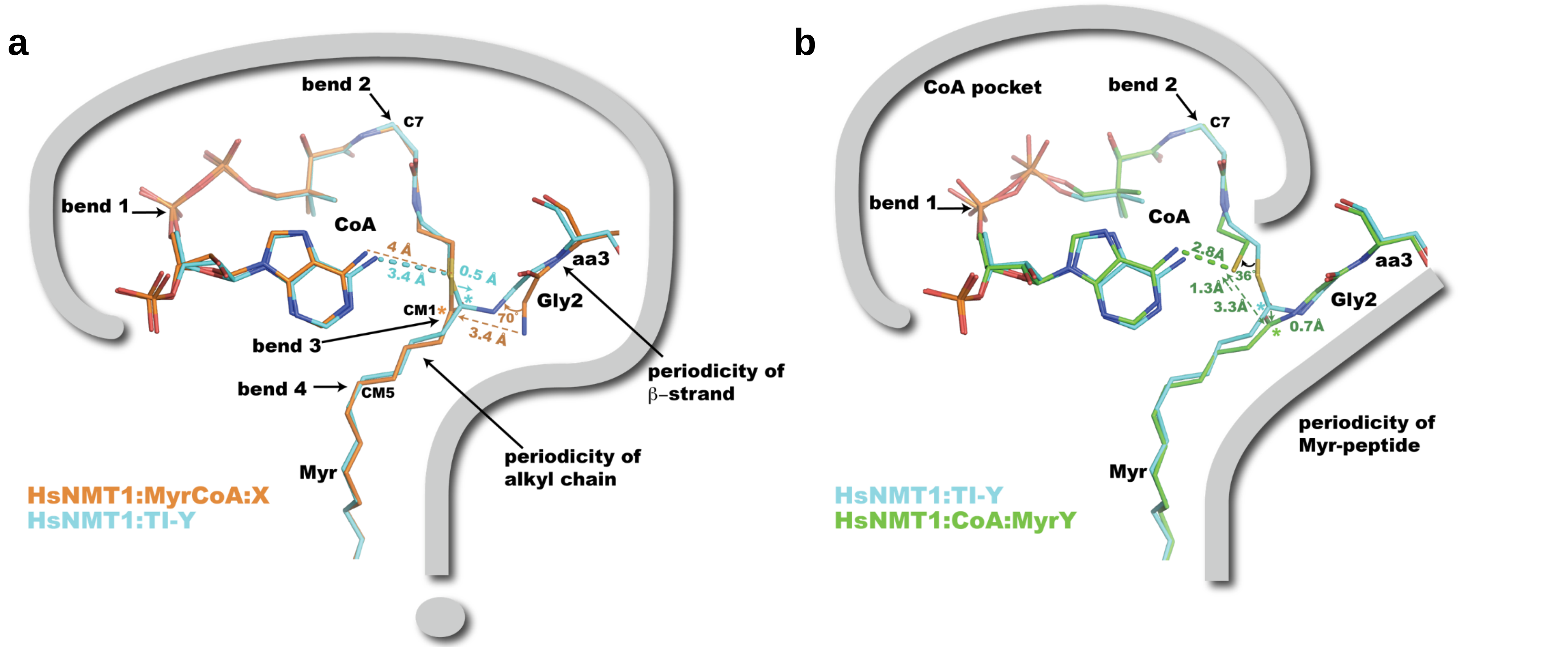

Supplementary Figure 5. Substrates and products changes along the catalytic reaction

a) Superimposition of HsNMT1:MyrCoA:X (orange carbons) and HsNMT1:TI-Y (cyan carbons) focusing on both substrate peptide and MyrCoA moieties reveals the 70˚ rotation of the N-terminal amino group from substrate peptide Gly2 towards MyrCoA CM1 carbonyl and the induced displacement of the latter to produce the tetrahedral intermediate. Carbon CM1 from the MyrCoA carbonyl is marked with a star. Bound MyrCoA presents a question mark-like shape with four previously reported bends bringing tension to the MyrCoA structure[3]. **b)** Superimposition of HsNMT1:TI-Y (cyan carbons) and HsNMT1:CoA:MyrY (cyan carbons) focused on TI-Y and CoA moieties reveals two motions at the level of the carbon of the newly formed amide group in the myristoylated product: (i) a back motion of this carbon in the amide plane that allows proper positioning of the carbonyl oxygen within the oxyanion hole, and (ii) a 0.7 Å shift of this electrophilic carbon away from the sulphur atom of CoA induced by the peptide β-strand conformation. This suppresses the third bend involved in the “question mark” shape of MyrCoA at its binding site. The newly formed thiolate anion moves 1.3 Å away from the amide bond of MyrY due to van der Waals repulsion with the newly formed N-C bond. This shift places the thiolate at an optimal distance to form an intramolecular H-bond with the amine group of CoA. However, despite rearrangements occurring upon breakdown of the reaction intermediate, the polarized carbonyl of the Myr-Gly2 amide group is 3.3 Å away from the leaving thiolate anion of CoA, a distance sufficient to lead to reformation of the TI via a 36˚ rotation around C2P/C3P bond of CoA.

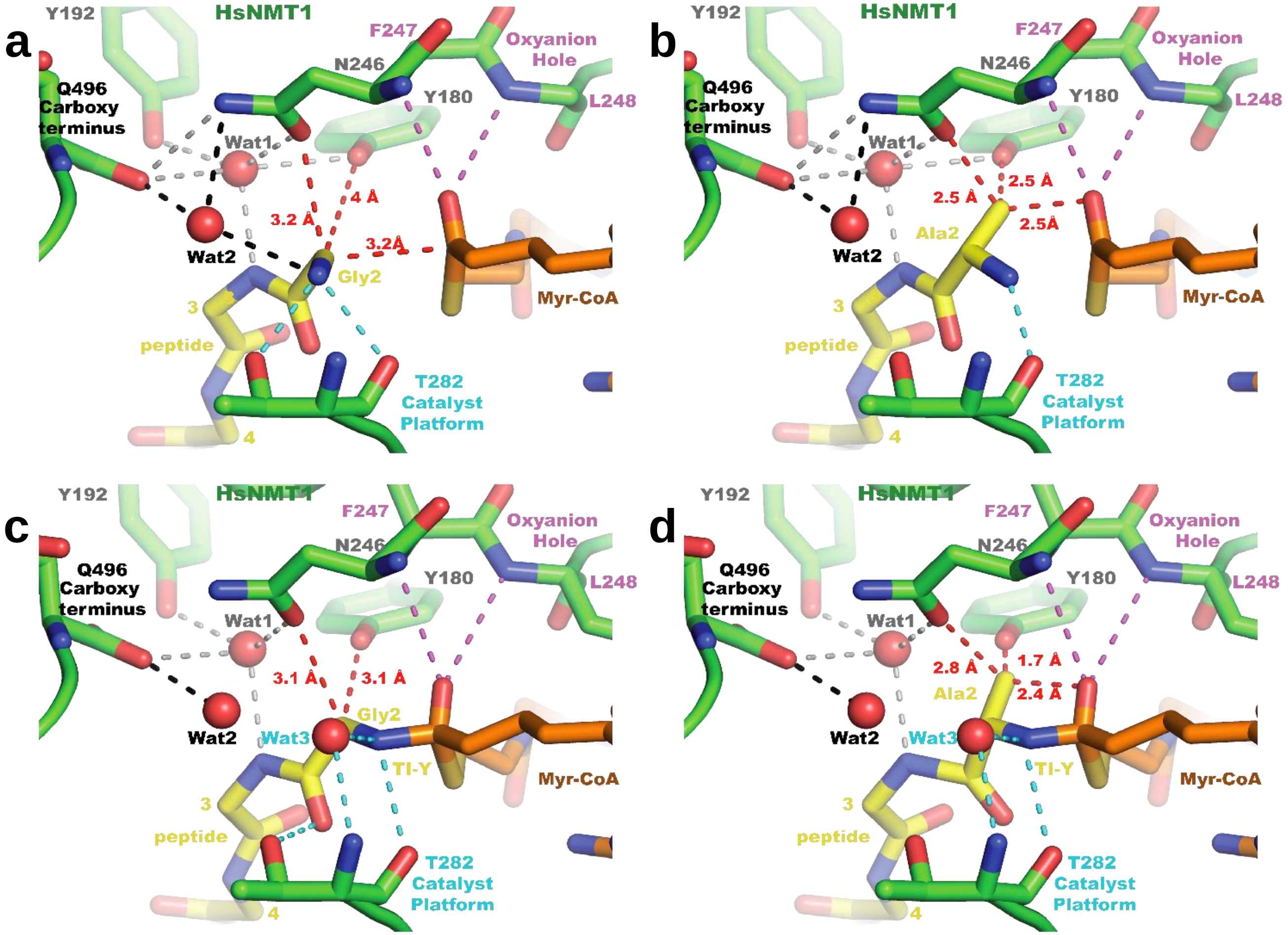

Supplementary Figure 6. Gly to Ala substitution prevents amino terminal rotation due to steric clashes

View of the HsNMT1 active site highlighting the Gly selectivity at position 2 of the substrate peptide constrained by both Asn246 and Tyr180 side chains together with the thioester bound **a)** substrates in HsNMT1:MyrCoA:X, **b)** substrates in HsNMT1:MyrCoA:X, in which Gly2 was replaced by Ala, **c)** the transition state in HsNMT1:TI-Y and **d)** the transition state in HsNMT1:TI-Y, in which Gly2 was replaced by Ala. The HsNMT1 chain is displayed as a green ribbon, and selected amino acids are shown as green sticks. Substrate peptides are shown as yellow sticks. MyrCoA and CoA moieties shown as orange sticks. Distances between either Ca of Gly2 or Cb of modelled Ala2 with both Asn246 and Tyr180 side chains and MyrCoA thioester are shown as red dashes. Hydrogen bond set1, linking Gly2 to the T282 catalyst platform, is shown as cyan dashes. Wat1-mediated hydrogen bond set2, linking the carboxy terminus of Q496 to hydroxyl groups of Tyr180/Tyr192 from the Ab-loop, amide group of substrate aa3, and amide of Asn246 are shown as grey dashes. Wat1-mediated hydrogen bond set3 linking the carboxy terminus of Gln496 to Gly2 ammonium are shown as black dashes. Water molecules from the solvent channel are shown as red spheres. Hydrogen bonds involved in the “oxyanion hole” are displayed as a magenta dashed line. The H-bond between thiolate and amine from CoA is shown with violet dashes.

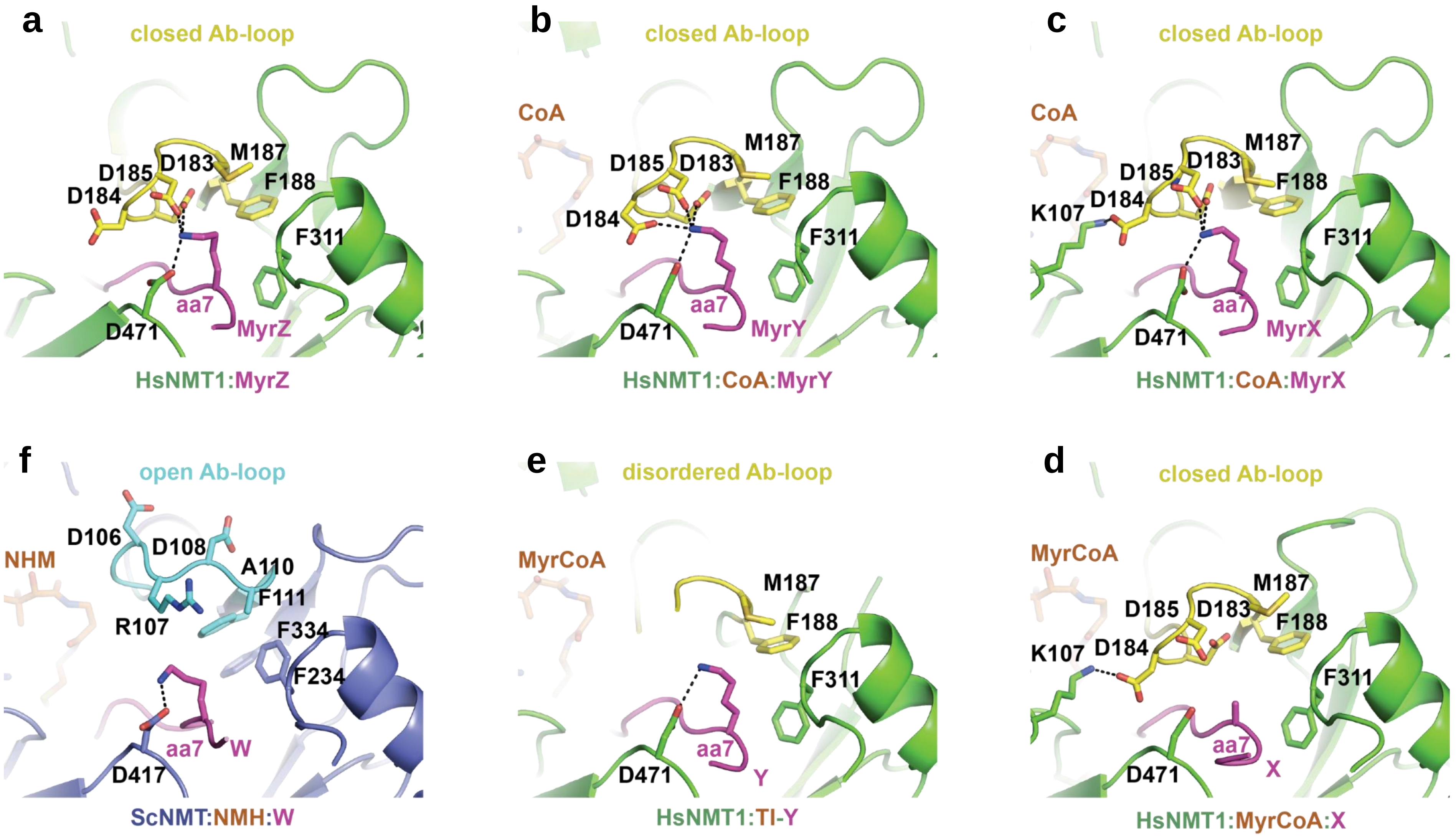

Supplementary Figure 7. Link between Ab-loop and aa7 substrate position

Outside view of the peptide substrate cavity allowing the accommodation of a Lys7 from the substrate peptide in the function of Ab-loop conformation. **a)** Products in HsNMT1:MyrZ, **b)** products in HsNMT1:CoA:MyrX, **c)** products in HsNMT1:CoA:MyrX, **d)** substrates in HsNMT1:MyrCoA:X, **e)** transition state in HsNMT1:TI-Y and, **f)** substrates in HsNMT1:NHM:W[4]. The HsNMT1 (green) and ScNMT (blue) main chains are in ribbons, and selected residues are in sticks. Main chain and selected residues of HsNMT1 Ab-loop (yellow) and ScNMT Ab-loop (cyan) are displayed as ribbon and sticks respectively. Substrate and product peptides are displayed as magenta ribbon, and selected amino acids are shown as magenta sticks. MyrCoA, CoA and NHM moieties are shown as orange sticks. Salt bridges are shown as black dashes.

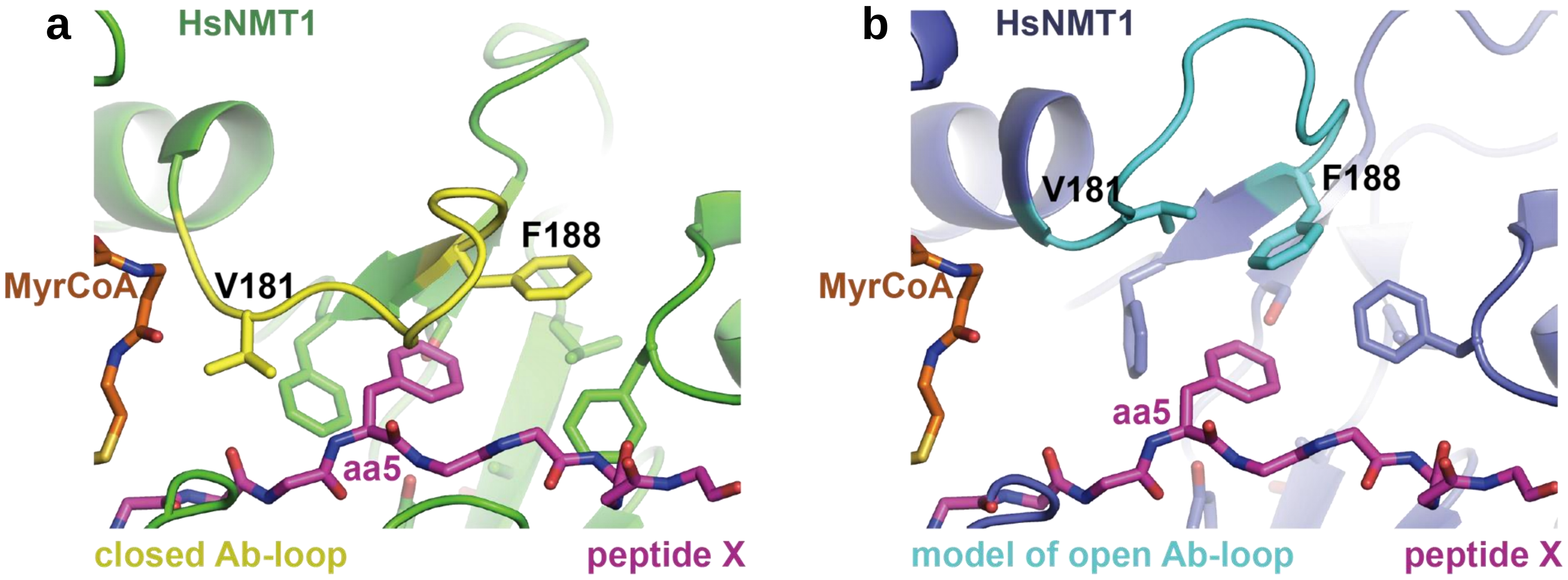

Supplementary Figure 8. Ab-loop conformation does not favour substrate aa5

Outside view of the peptide substrate cavity that allows accommodation of aa5 from the substrate peptide in the function of Ab-loop conformation. **a)** Substrates in HsNMT1:MyrCoA:X harbouring a closed conformation of the Ab-loop. The HsNMT1 chain is displayed as a green ribbon, and selected amino acids are shown as green sticks. The closed conformation of the Ab-loop is displayed as a yellow ribbon, and selected amino acids are shown as yellow sticks. **b)** Substrates in HsNMT1:MyrCoA:X, in which the Ab-loop was modelled in an open conformation based on the ScNMT:NHM:W crystal structure[4]. The HsNMT1 chain is displayed as a blue ribbon, and selected amino acids are shown as blue sticks. The model of the open conformation of the Ab-loop is displayed as a cyan ribbon, and selected amino acids are shown as cyan sticks.

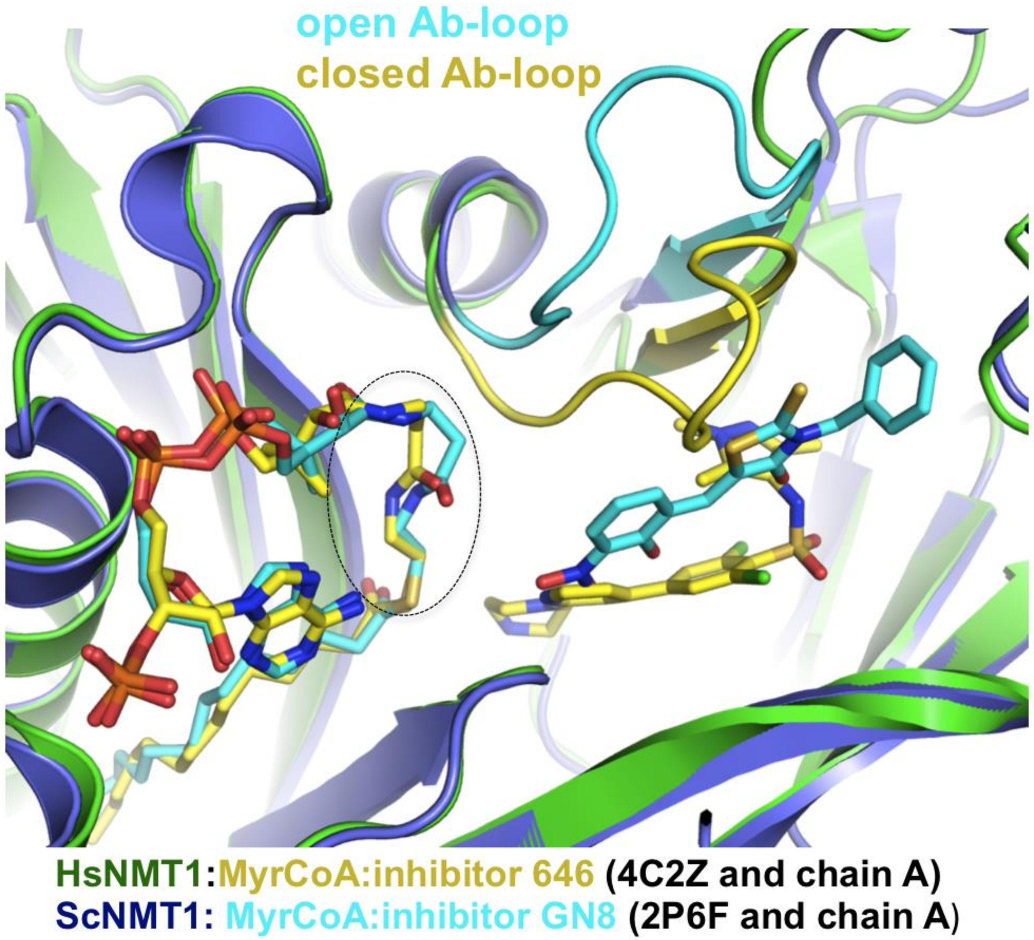

Supplementary Figure 9. NMT:MyrCoA:inhibitor complexes show that the MyrCoA compaction depends only on the Ab-loop conformation
Structural superimposition of the ternary ScNMT:MyrCoA:inhibitor GN8 complex (PDB 2P6F, blue)[2] and the ternary HsNMT1:MyrCoA:inhibitor 646 (PDB 4C2Z, green)[6] complex shows that the Ab-loop closed conformation (yellow) observed in the latter induced MyrCoA compaction (black dashed line circle) in comparison to MyrCoA (cyan) from ScNMT:MyrCoA. GN8 and 646 inhibitors are shown in cyan and yellow, respectively.

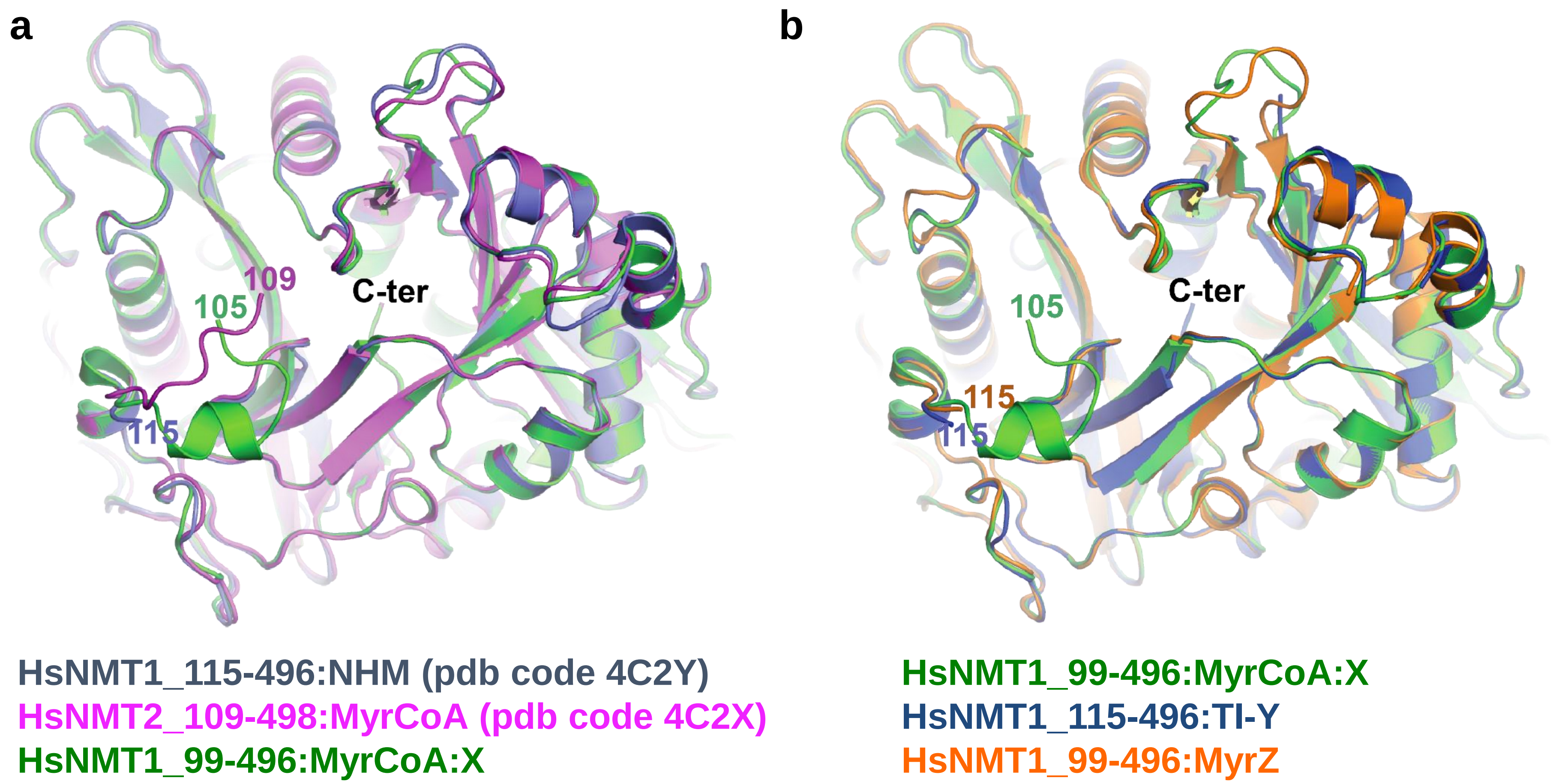

Supplementary Figure 10. Comparison of solved structures and previous structures of HsNMT

a) Structural superimposition of the previously solved binary HsNMT1:NHM complex (PDB 4C2Y, blue ribbon)[6] and the binary HsNMT2:MyrCoA complex (PDB 4C2Y, magenta ribbon)[6] with the ternary HsNMT1:MyrCoA:X complex solved in this study (green ribbon) shows the importance of residues 100-108 in the formation of a folded N-terminal B′A′-loop. **b)** Structural superimposition of the ternary HsNMT1:MyrCoA:X complex (green ribbon), ternary HsNMT1:TI-Y complex (blue ribbon), and binary HsNMT1:MyrZ complex (orange ribbon) shows that CoA release induced disordering of B′A′-loop.

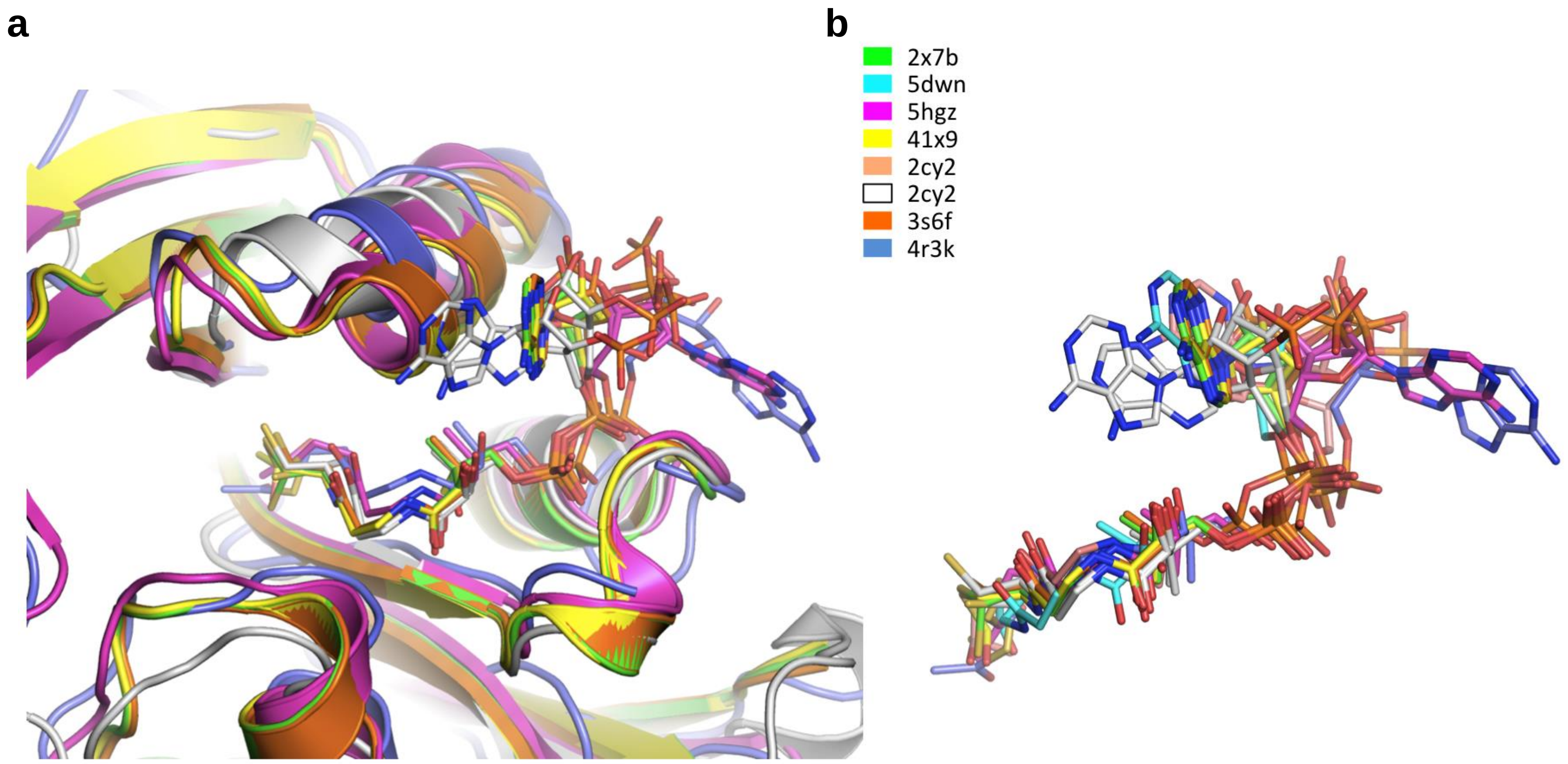

Supplementary Figure 11. Structural alignment of AcCoA/CoA-bound NAT structures suggesting that adenosine moiety flexibility could be important for CoA release from NMT active site

a) The different NATs structures were structurally aligned based on their conserved GNAT domains. Chains are displayed as ribbons with their bound AcCoA or CoA bound shown as sticks coloured according to their PDB codes. **b**) Focus on the CoA/AcCoA moieties reveals the flexibility of the adenine ring.

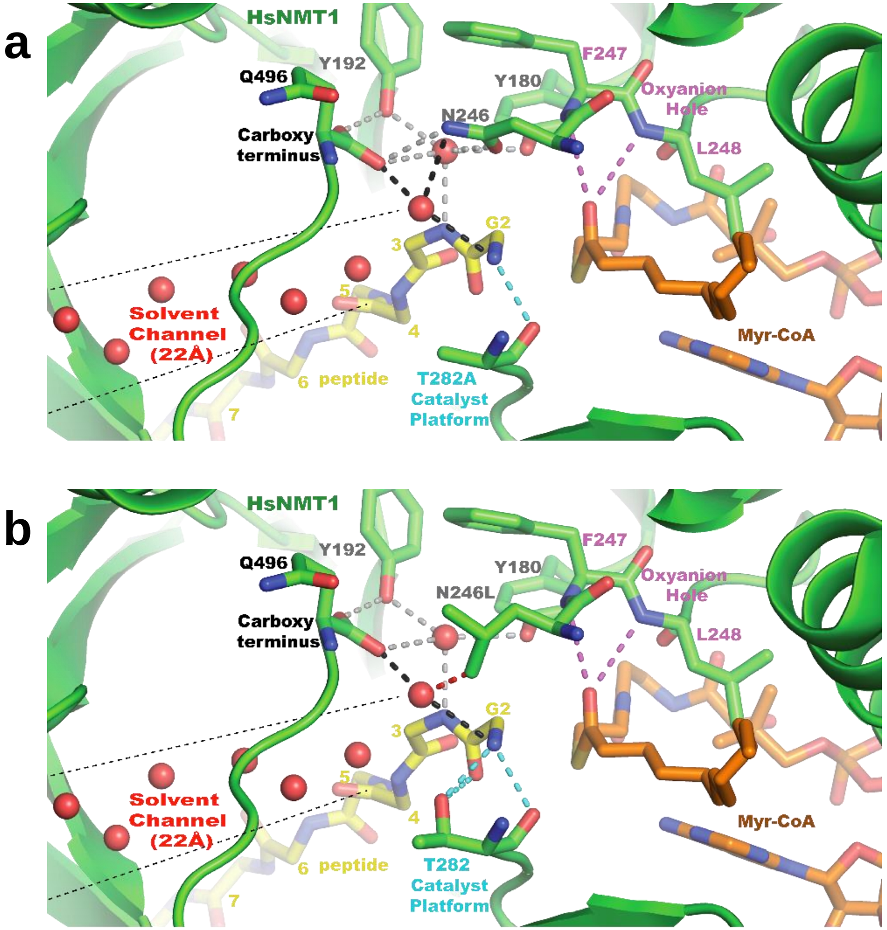

Supplementary Figure 12. Structural impact of either T282A or N248L substitution modelled on HsNMT1 active site. View of the HsNMT1 active site of the HsNMT1:MyrCoA:X ternary complex in which **a)** Thr282Ala substitution was modelled **b)** Asn246Leu substitution was modelled. In ScNMT, single amino acid mutants Thr205Ala (T282 in HsNMT1) and Asn169Leu (Asn246 in HsNMT1) targeting the catalysis platform and the first H-bond set 1 are consistent with our newly proposed catalytic mechanism[4]. Both two-fold k_{burst} and three-fold K_m decreases in ScNMT-Thr205Ala are in agreement with the moderate effect expected from the analogous Thr282Ala substitution, which was modelled with HsNMT1. This substitution indeed removes two out of three H-bonds made by Gly2 of X peptide. Similarly, the Asn248Leu substitution in HsNMT1 mimicking Asn169Leu in ScNMT reveals that this mutation would disturb both the water-mediated interaction of the carboxy terminus of Gly2 in peptide X and H-bond set 1, involved in optimal positioning of the C-terminus of the enzyme and peptide X in the catalytic centre. These effects cause both a 10-fold reduction of the k_{burst} and of the K_m value measured with ScNMT.

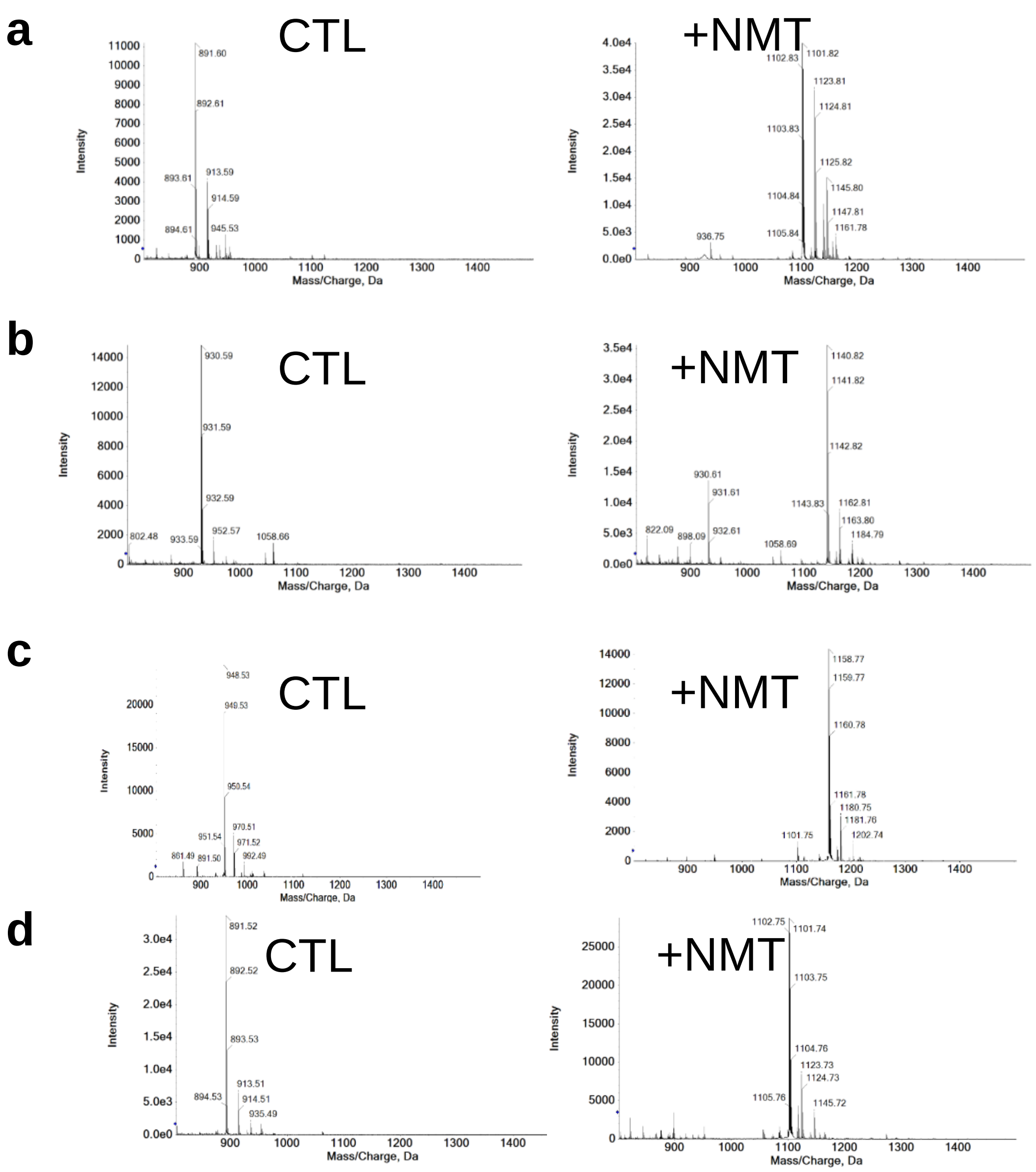

Supplementary Figure 13. Mass spectrometry analysis of NMT-catalysed MYR of N-Lys-containing peptides
Both control MS1 spectra of the four peptides described in Fig. 8 in the absence (left) or presence of NMT (right) are displayed as follows: **a)** GKVLSKIF (Arf6), **b**) GKQNSKLR (HPCA), **c**) AcGKSFSKPR, and **d**) AcKSFSKPR. The MYR mass shift is 210 Da.

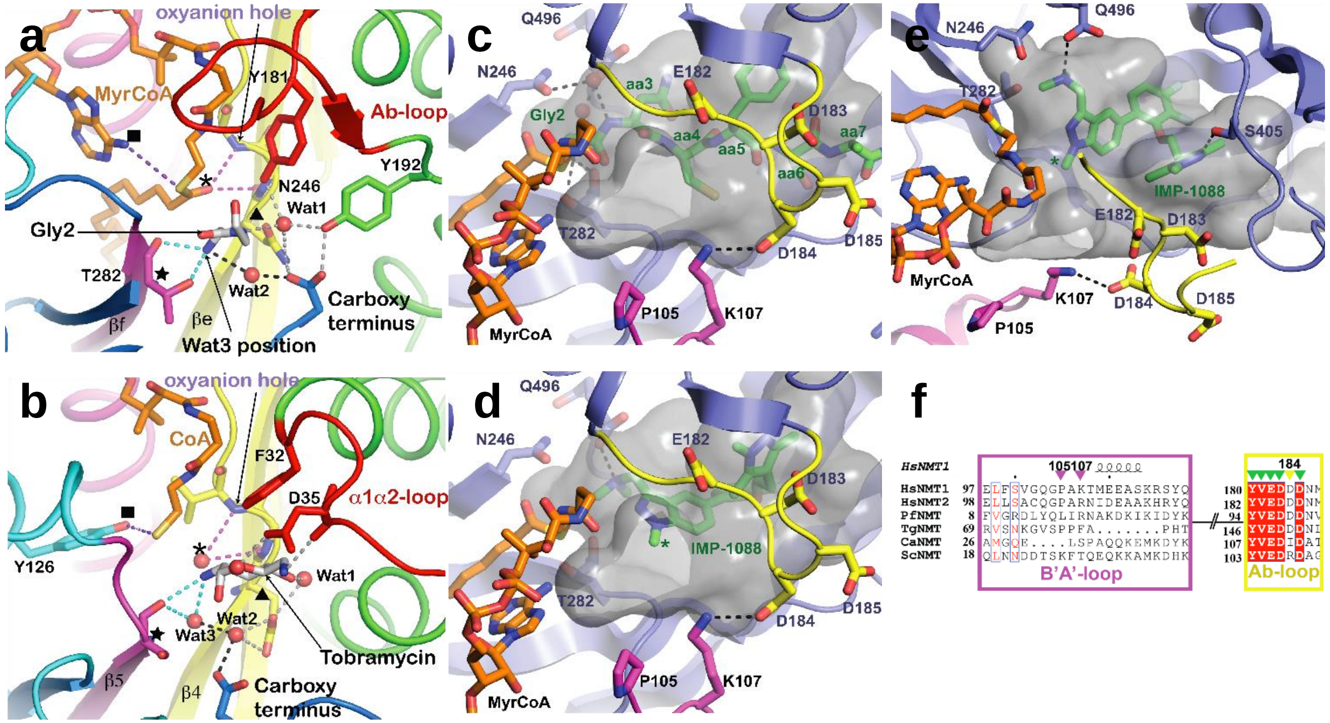

Supplementary Figure 14. Transposition of the NMT mechanism to GNAT proteins and inhibitor selectivity.
Comparison of **a)** HsNMT1:MyrCoA:X and **b)** aminoglycoside 2'N-acetyltransferase from *M. tuberculosis* (AgNAT) bound to CoA and the inhibitor tobramycin reveals conservation of the catalytic mechanism of HsNMT1 with other GNAT enzymes. The carbonyl of the residue positioned at the end of β5 of the aminoglycoside 2'N-acetyltransferase (black star) is likely to act as a catalytic platform, while the residue positioned at the end of β4 of the same enzyme is likely to organize the wat1/wat2 network involved in ammonium group deprotonation. The oxyanion hole bulge and thioester carbonyl from AcCoA position are also conserved, as shown by the pink dashes and black star, respectively. The α1α2-loop in the GNAT superfamily is the equivalent of the Ab-loop in HsNMT1 and is coloured in red. The hydroxyl group of the Tyr126 residue in AgNAT, suggested to act as an acid to reprotonate the thiolate anion, is placed at an equivalent position of the identified CoA amine group to stabilise the leaving thiolate group as shown by the violet dashes. Secondary structure colour code of the GNAT catalytic domain is as in **Figure 1b**. Peptide X and tobramycin are shown partially as grey sticks, for clarity. MyrCoA and CoA moieties are shown as orange sticks. Hydrogen bond set1, set2, and set3 identified in HsNMT1 and AgNAT are shown as cyan, grey, and dark dashes. Wat1, 2 and 3 molecules are shown as red spheres. A detailed comparison of the HsNMT1 active site around the B'A'-loop and Ab-loop interaction in **c)** HsNMT1:MyrCoA:X, **d)** and **e)** HsNMT1:MyrCoA:X, in which the X substrate peptide has been replaced by IMP-1088, showing that the IMP-1088 inhibitor binds the highly conserved peptide-binding site shown in two different views. Grey surface of the peptide-binding site shows that there is a space not occupied by the inhibitor close to the B'A'-loop and Ab-loop interaction. A green asterisk highlights the moiety of IMP-1088 that might be used for selectivity improvement. **f)** Sequence alignment of NMT proteins obtained with ESPript3 (http://espript.ibcp.fr,[5]) with the corresponding secondary structure of the HsNMT1:MyrCoA:X, reveals that the B'A'-loop/Ab-loop interaction motif is not conserved among NMTs. Residues in white and red indicate identical and conserved residues, respectively. Magenta and yellow broken boxes indicate the residues participating in the B'A'-loop defined in the ScNMT:MyrCoA structure and Ab-loop as defined in the ScNMT and CaNMT crystal structures, respectively. Residues identified in this study important for the MYR mechanism are indicated with triangles. Green triangle defines conserved residues, whereas yellow and magenta triangles denote non-conserved ones using B'A' and Ab-loop colour coding.

Supplementary Tables

Supplementary Table 1. Data collection and refinement statistics concerning wild type NMT1 in complex with peptides X, Y, U and V.

PDB accession code	6QRM	6EHJ	6SJZ	6SK2
Crystal molecules	HsNMT1: MyrCoA:X	HsNMT1: MyrCoA:Y	HsNMT1: MyrCoA:U	HsNMT1: MyrCoA:V
Chain A ligands	MyrX	TI-Y/MyrY	U	MyrV
Chain B ligands	X	MyrY	U	V
Peptide sequence	GNCSFSKRRA	GSNKSKPK	AcGKSFSKPR	AcKSFSKPR
Space group	$P2_12_12$	$P2_12_12$	$P2_12_12$	$P2_12_12$
Number of complexes in ASU	2	2	2	2
Unit cell (Å)	a= 79.44<br>b=178.93<br>c= 58.41	a=80.25<br>b=177.54<br>c=58.14	a= 79.91<br>b=178.90<br>c=58.38	a= 78.82<br>b= 178.44<br>c= 58.27
Wavelength (Å)	0.96770	0.97625	0.96600	0.98400
Beamline	ID30a3 (ESRF)	I03 (DLS)	ID30a1 (ESRF)	PX1 (SOLEIL)
Resolution range (Å)	47.77-2.30 (2.38-2.30)	47.08-2.1 (2.15-2.10)	48.89-1.99 (2.05-1.99)	48.79-1.90 (1.94-1.90)
R_{merge}	0.143 (0.669)	0.112 (0.968)	0.103 (0.968)	0.102 (0.941)
R_{pim}	0.051 (0.226)	0.047 (0.533)	0.038 (0.203)	0.028 (0.252)
Number of total reflections	348468 (33889)	330247 (14866)	461333 (26772)	930363 (61061)
Number of unique reflections	37748 (3619)	49357 (3595)	57674 (3937)	65710 (4177)
Mean ([I]/SD[I])	12.8 (3.6)	12.3 (1.3)	14.4 (4)	15.4 (3.2)
$CC_{1/2}$	0.996 (0.875)	0.998 (0.508)	0.998 (0.508)	0.999 (0.934)
Completeness (%)	99.7 (99.8)	99.8 (99.3)	99.5 (93.6)	99.9 (99.7)
Multiplicity	9.2 (9.4)	6.7 (4.1)	8 (6.8)	14.2 (14.6)
Wilson plot B ($Å^2$)	29.09	10.4	21.32	26.6
Number of atoms	7003	6609	7209	7411
Protein	6334	5982	6347	6422
Peptide	67		114	57
MyrCoA	63		126	63
MyrCoA-peptide		122		
Myr-peptide	69	148		76
CoA	48	96		48
Ions / Glycerol	2 Cl^-, 24	24	2 Cl^-, 24	0, 18
Water	396	237	596	727
R_{work} (%)	17.20	18.43	17.16	17.20
R_{free} (%)	20.33	23.86	20.16	22.00
<B > ($Å^2$)	30.7	37.83	27.1	31.6
rms bond length deviation (Å)	0.009	0.008	0.004	0.006
rmsd angle deviation (°)	0.989	0.979	0.810	0.858

Ac is for N-acetyl.

Values in parentheses correspond to the highest resolution shell.
$R_{merge} = \Sigma(I_{hl} - < I_h >)/\Sigma < I_h >$
$R_{meas} = \Sigma\sqrt{}(n_h/n_h - 1)(I_{hl} - < I_h >)/\Sigma < I_h >$
$R_{pim} = \Sigma\sqrt{}(1/n_h - 1)(I_{hl} - < I_h >)/\Sigma < I_h >$

Supplementary Table 2. Data collection and refinement statistics of NMT1 variants

PDB accession code	6SKJ	6SK8	6SK3
Crystal molecules	ΔC2:MyrCoA:S	ΔC3:MyrCoA:T	ΔC3:MyrCoA:S
Chain A	S/TI-S	T	S
Chain B	TI-S/Myr-S	T	S
Peptide sequence	GNCFSKPR	GDCFSKPR	GNCFSKPR
Space group	C121	C121	C121
Number of complexes in	2	2	2
Unit cell (Å)	*a*=92.97<br>*b*=58.27<br>*c*=154.13<br><br>β=90.92°	a= 92.62<br>b=58.26<br>c= 154.79<br><br>β=91.26°	a= 91.81<br>b=58.07<br>c= 148.21<br><br>β=92.25°
Wavelength (Å)	0.978565	0.978565	0.978565
Beamline	PX1 (Soleil)	PX1 (Soleil)	PX1 (Soleil)
Resolution range (Å)	49.37-2.8 (2.95-2.80)	49.31-1.87 (1.91-	49.07-2.70 (2.83-
R_{merge}	0.122 (0.885)	0.159 (1.219)	0.171 (0.707)
R_{pim}	0.053 (0.393)	0.063 (0.467)	0.074 (0.322)
Number of total reflections	114615 (4601)	507596 (33696)	129478 (4104)
Number of unique	19085 (706)	68387 (4360)	21519 (630)
Mean ([I]/SD[I])	10.8 (2.0)	7.3 (1.6)	7.4 (2.0)
$CC_{1/2}$	0.997 (0.796)	0.994 (0.665)	0.994 (0.842)
Completeness (%)	92.5 (79.4)	99.9 (100)	99.1 (95.8)
Multiplicity	6.0 (5.4)	7.4 (7.7)	6.0 (5.0)
Wilson plot B (Å^2)	36.12	25.54	38.84
Number of atoms	6673	7493	6514
Protein	6286	6338	6234
Peptide	31	126	126
MyrCoA	63	126	126
MyrCoA-peptide	122		
Myr-peptide	38		
CoA	24		
Ions / Glycerol	1 Mg^{2+}, 18	1 Cl^-, 36	1 Mg^{2+}, 12
Water	90	843	15
R_{work} (%)	18.90	20.78	24.59
R_{free} (%)	21.88	24.39	26.86
<B > (Å^2)	29.6	30.2	39
rms bond length deviation	0.003	0.003	0.004
rmsd angle deviation (°)	0.738	0.691	0.768

Values in parentheses correspond to the highest resolution shell.

$R_{merge} = \Sigma(I_{hl} - < I_h >)/\Sigma < I_h >$

$R_{meas} = \Sigma\sqrt{}(n_h/n_h - 1)(I_{hl} - < I_h >)/\Sigma < I_h >$

$R_{pim} = \Sigma\sqrt{}(1/n_h - 1)(I_{hl} - < I_h >)/\Sigma < I_h >$

Supplementary Table 3. Oligonucleotide primers used in this study.

Variant		Primer sequence
DeltaC2	For	5'-CGAGAATCTTTA TTTTCAGGGCGGATCCGAATTCTCAGTGGGTCAGGGACCT-3'
	Rev	5'-GGTGGTGCTCGAGTTATTGTTACACCAGTCCAACCTTCTCTGC-3'
DeltaC3	For	5'-CGAGAATCTTTA TTTTCAGGGCGGATCCGAATTCTCAGTGGGTCAGGGACCT-3'
	Rev	5'-GTGGTGCTCGAGTTATTGTAGTTACAGTCCAACCTTCTCTGCCC-3'
Y180A	For	5'-CTCCTGAATGAGAACGCTGTGGAAGATGATGA-3'
	Rev	5'-TCATCATCTTCCACAGCGTTCTCATTCAGGAG-3'
Y180P	For	5'-CACCCTCCTGAATGAGAACCCGGTGGAAGATGATGACACCAT G -3'
	Rev	5'-GTTGTCATCATCTTCCACCGGGTTCTCATTCAGGAGGGTG-3'
V181A	For	5'-TGAATGAGAACTATGCGGAAGATGATGACAA-3'
	Rev	5'-TTGTCATCATCTTCCGCATAGTTCTCATTCA-3'
Y181L	For	5'-CTGAATGAGAACTATCTGGAAGATGATGACA-3'
	Rev	5'-TGTCATCATCTTCCAGATAGTTCTCATTCAG-3'
Y192A	For	5'-ATGTTCCGATTTGATGCTTCCCCGGAGTTTCT-3'
	Rev	5'-AGAAACTCCGGGGAAGCATCAAATCGGAACAT-3'
K107E	For	5'-GGTCAGGGACCTGCCGAAACCATGGAGGAGGC-3'
	Rev	5'-GCCTCCTCCATGGTTTCGGCAGGTCCCTGACC-3'
N246A	For	5'-GAAGAAGATGGTAGAGATCGCGCTTCTTGTGTGTCCACAAG-3'
	Rev	5'-CTTGTGGACACACAGGAAGGCGATCTCCACCATCTTCTTC-3'
K252E	For	5'-CAACTTCCTGTGTGTCCACGAGAAGCTGCGTTCCAAGAG-3'
	Rev	5'-CTCTTGGAACGCAGCTTCTCGTGGACACACAGGAAGTTG-3'

Supplementary Methods

Production of site-directed NMT1 variants

C-terminal DeltaC2 and DeltaC3 truncation of Long-HsNMT1 were generated by PCR using primers reported in **Supplementary Table 3.** The resulting PCR products were subcloned in the pET28 derivative plasmid using Fast digest BamH1 and XhoI restriction enzymes (Thermo Fisher Scientific, Waltham, MA). Point mutants of long-HsNMT1 were made using a QuikChange site-directed mutagenesis kit (Stratagene, San Diego, CA) using primers pairs displayed in **Supplementary Table 3**.

C-terminal truncation and points mutants of long-HsNMT1 were expressed, purified and crystallized following the same protocol as the WT protein. Truncations of Long-HsNMT1 were expressed and purified as previously described[7]. Suitable crystals of ΔC2*:MyrCoA:S,* ΔC3*:MyrCoA:S* and ΔC3*:MyrCoA:T* were obtained by co-crystallization using the hanging-drop vapor diffusion method at 20°C. Crystallization droplets were formed by mixing 2 µL of the of HsNMT1:MyrCoA:peptide complex (ratio 1:1.5:1.5) at 7.5 mg/mL (i.e., ~160/250/250 µM) with 2 µL of the precipitant solution containing 0.1 M $MgCl_2$, 0.2 M NaCl, 0.1 M sodium citrate pH 5.6, and 18-24% (w/v) PEG 6K or 8K. Crystals were cryoprotected in the reservoir solution supplemented with 15% (v/v) glycerol and flash cooled in liquid nitrogen. Complete X-ray dataset of ΔC2*:MyrCoA:S,* ΔC3*:MyrCoA:S* and ΔC3*:MyrCoA:T* were collected at λ = 0.978565 from a single crystal at 100*K* in the Proxima1 beamline, at the French National Synchrotron Facility (SOLEIL).

Datasets were integrated with XDS[8] and scaled and reduced using AIMLESS from the CCP4 package[9]. Crystals belonged to the space group C121 with similar unit cell parameters (summarized in **Supplementary Table 2**) and contained two NMT molecules per asymmetric unit. Structure resolution was accomplished in all cases

using the molecular replacement method. The different structure were solved using PHASER[10] and protein coordinates of a Long-HsNMT1 model (PDB entry 509V) as a search model. Structures were subjected to alternating refinement cycles using PHENIX and manual model building using COOT[11, 12, 13]. NCS restrictions were applied. In both structures, the electron density maps allowed us to model most of both HsNMT1 molecules of the asymmetric unit. The good quality of the electron density maps also enabled the refinement of substrate peptide, reaction intermediate, and reaction product molecules bound to HsNMT1 in each complex. In the refinement of the ΔC2*:MyrCoA:S,* ΔC3*:MyrCoA:S* and ΔC3*:MyrCoA:T* complexes, chemical compound libraries were generated using PRODRG server[14] in combination with eLBOW from the PHENIX suite. The geometry of the final models was validated using MOLPROBITY[15]. X-ray data collection and refinement statistics are summarized in **Supplementary Table 2**.

Supplementary References

1. Weston SA*, et al.* Crystal structure of the anti-fungal target N-myristoyl transferase. *Nat Struct Biol* **5**, 213-221 (1998).

2. Wu J, Tao Y, Zhang M, Howard MH, Gutteridge S, Ding J. Crystal structures of Saccharomyces cerevisiae N-myristoyltransferase with bound myristoyl-CoA and inhibitors reveal the functional roles of the N-terminal region. *J Biol Chem* **282**, 22185-22194 (2007).

3. Bhatnagar RS*, et al.* Structure of N-myristoyltransferase with bound myristoylCoA and peptide substrate analogs. *Nat Struct Biol* **5**, 1091-1097 (1998).

4. Farazi TA, Waksman G, Gordon JI. Structures of Saccharomyces cerevisiae N-myristoyltransferase with bound myristoylCoA and peptide provide insights about substrate recognition and catalysis. *Biochemistry* **40**, 6335-6343 (2001).

5. Robert X, Gouet P. Deciphering key features in protein structures with the new ENDscript server. *Nucleic Acids Res* **42**, W320-324 (2014).

6. Thinon E*, et al.* Global profiling of co- and post-translationally N-myristoylated proteomes in human cells. *Nat Commun* **5**, 4919 (2014).

7. Castrec B*, et al.* Structural and genomic decoding of human and plant myristoylomes reveals a definitive recognition pattern. *Nat Chem Biol* **14**, 671-679 (2018).

8. Kabsch W. Automatic processing of rotation diffraction data from crystals of initially unknown symmetry and cell constants. *J Appl Cryst* **26**, 795-800 (1993).

9. Evans PR, Murshudov GN. How good are my data and what is the resolution? *Acta Crystallogr D Biol Crystallogr* **69**, 1204-1214 (2013).

10. McCoy AJ, Grosse-Kunstleve RW, Adams PD, Winn MD, Storoni LC, Read RJ. Phaser crystallographic software. *J Appl Crystallogr* **40**, 658-674 (2007).

11. Murshudov GN*, et al.* REFMAC5 for the refinement of macromolecular crystal structures. *Acta Crystallogr D Biol Crystallogr* **67**, 355-367 (2011).

12. Adams PD*, et al.* PHENIX: a comprehensive Python-based system for macromolecular structure solution. *Acta Crystallogr D Biol Crystallogr* **66**, 213-221 (2010).

13. Emsley P, Lohkamp B, Scott WG, Cowtan K. Features and development of Coot. *Acta Crystallogr D Biol Crystallogr* **66**, 486-501 (2010).

14. Schuttelkopf AW, van Aalten DM. PRODRG: a tool for high-throughput crystallography of protein-ligand complexes. *Acta Crystallogr D Biol Crystallogr* **60**, 1355-1363 (2004).

15. Chen VB*, et al.* MolProbity: all-atom structure validation for macromolecular crystallography. *Acta Crystallogr D Biol Crystallogr* **66**, 12-21 (2010).
