# Supplementary material for: High-resolution snapshots of human N-myristoyltransferase in action illuminate a mechanism promoting N-terminal Lys and Gly myristoylation
Source: Nat Commun. 2020 Feb 28;11:1132. doi: 10.1038/s41467-020-14847-3 (PMC7048800; doi:10.1038/s41467-020-14847-3)
Supplement: Supplementary file 3 — Description of Additional Supplementary Files [file 41467_2020_14847_MOESM3_ESM.pdf]

Description of Additional Supplementary Files

File Name: Supplementary Movie 1

Description: Cartoon of the catalytic mechanism
